# TAMIS J-pouch excision

**DOI:** 10.1007/s10151-018-1905-z

**Published:** 2018-12-12

**Authors:** A. Caycedo-Marulanda, G. Ma

**Affiliations:** 1 Health Sciences North, 65 Larch St. Suite 308, Sudbury, ON P3E 1B8 Canada; 2 Northern Ontario School of Medicine, Sudbury, ON Canada; 3 Colorectal Surgery North, Sudbury, ON Canada

The ileal pouch anal anastomosis (IPAA) was introduced in 1978 by Parks and Nicholls. The procedure was designed for patients with ulcerative colitis, for whom a total proctocolectomy was indicated, as an alternative to a permanent ileostomy [1]. IPAA has become the gold standard for patients with chronic ulcerative colitis and familial adenomatous polyposis. This procedure carries some risks, including failure of the pouch.

Short-term morbidity after IPAA ranges from 28 to 58% and failure is due to technical or inflammatory complications. Remzi et al. have published the largest series on redo pouch surgery [2]: according to the authors, leak/fistula, obstruction, dysfunction, pelvic abscess and pouchitis are the top five indications for redo surgery. All patients who had revisions or redo pouch procedures required a laparotomy, even if the original operation was performed using minimally invasive techniques.

Redo bowel surgery is not easy .This is particularly evident in the pelvis. Pouch excision carries a high rate of short- and long-term complications, 57% and 37%, respectively [3].

The literature on minimally invasive approaches for redo IPAA is very limited. In 2016, Chadi et al. submitted a presentation to the annual Society of American Gastrointestinal and Endoscopic Surgeons (SAGES) meeting with a case series of seven patients undergoing laparoscopic revision of their pouch with two patients having laparoscopic pouch excision.

The novel technique of transanal total mesorectal excision (taTME) was originally described by Sylla et al. [4]. This technically challenging technique is associated with favorable results in the management of rectal neoplasia and is becoming popular. TaTME has even been used for the initial construction of IPAA. This approach has been demonstrated to have a significant learning curve. The need to learn the anatomical landmarks in the bottom-up fashion and the potential for devastating complications has also led authors to caution centers and surgeons on the careful and selective implementation of the technique [5].

We present a case of an adult female with a long history of UC, refractory to medical therapy who underwent a laparoscopic total proctocolectomy and J-pouch reconstruction at our center. Unfortunately after the initial discharge from hospital, the patient required multiple admissions for pelvic sepsis. Ultimately, she was re-referred internally for a second opinion after the clear evidence of J-pouch failure secondary to an anastomotic leak. During the last admission, it was decided to bring her to the operating room, semi-electively for a TAMIS J-pouch excision. Prior to the procedure she was optimized medically.

The operation was started transanally, using the TAMIS platform. The anastomosis was carefully dismantled, followed by cephalad dissection of the pouch. Specific attention to plane identification was observed. After most of the dissection was completed from the bottom up, a second team joined for laparoscopic assistance, sharp dissection was conducted and eventually the rendezvous occurred. The pouch was completely excised from the pelvis. A Pfannenstiel incision was created and the pouch was then exteriorized and divided. A new pouch was constructed and subsequently placed in the abdomen. Further dissection of small adhesions was performed laparoscopically to ensure adequate reach. The new pouch was laid in the pelvis, to be guided transanally into the deep pelvis. A hand sewn anastomosis was then performed using interrupted stitches of 2-0 Vicryl^®^. The final step was to re-model the diverting loop ileostomy (see video link).

The patient recovered uneventfully on the surgical ward and was discharged on postoperative day 3. The loop ileostomy was reversed at 8 weeks, after satisfactory endoscopic and radiologic assessments of pouch integrity.

To our knowledge, we present the first description of the transanal approach as an alternative for difficult IPAA excision. It is possible that the transanal approach provides a less hostile field, for the surgeon to re-enter the pelvis, when compared with a purely transabdominal approach. Perhaps the benefits of minimally invasive surgery can still serve the patient. It is imperative that surgeons attempting this procedure have an extensive experience in IPAA surgery and taTME approach.

## Electronic supplementary material

Below is the link to the electronic supplementary material.

Supplementary material 1 (MP4 370812 KB)

## References

[1] Parks A, Nicholls R (2018). Proctocolectomy without ileostomy for ulcerative colitis. Br Med J.

[2] Remzi FH, Aytac E, Ashburn J, Gu J, Hull TL, Dietz DW (2015). Transabdominal redo ileal pouch surgery for failed restorative proctocolectomy. Ann Surg.

[3] Lightner AL, Dattani S, Dozois EJ, Moncrief SB, Pemberton JH, Mathis KL (2017). Pouch excision: indications and outcomes. Colorectal Dis.

[4] Sylla P, Rattner DW, Delgado S, Lacy AM (2010). NOTES transanal rectal cancer resection using transanal endoscopic microsurgery and laparoscopic assistance. Surg Endosc.

[5] Atallah SB, DuBose AC, Burke JP, Nassif G, deBeche-Adams T, Frering T (2017). Uptake of transanal total mesorectal excision in North America. Dis Colon Rectum.

